# Climate change and challenges for health surveillance in the Oropouche emergency

**DOI:** 10.1590/0034-7167.202578suppl302

**Published:** 2025-10-27

**Authors:** Cintia Lepaus Thomas, Karllian Kerlen Simonelli Soares, João Paulo Cola, Ethel Leonor Noia Maciel

**Affiliations:** I Universidade Federal do Espírito Santo, Epidemiology Laboratory. Vitória, Espírito Santo, Brazil; II Prefeitura Municipal de Alfredo Chaves, Municipal Health Department. Alfredo Chaves, Espírito Santo, Brazil; III Universidade Federal do Espírito Santo, Graduate Program in Public Health. Vitória, Espírito Santo, Brazil; IV Secretaria de Estado da Saúde do Espírito Santo. Vitória, Espírito Santo, Brasil; V Universidade Federal do Espírito Santo, Department of Nursing. Vitória, Espírito Santo, Brazil

The year 2024 was marked by a significant increase in Oropouche cases in Brazil, with widespread reports in states outside the traditional endemic area of the Legal Amazon. Espírito Santo, like other states in the Southeast region, began reporting indigenous cases, attracting the attention of national and international health authorities. This geographic expansion of the Oropouche virus, transmitted primarily by the insect *Culicoides paraensis*, raises important discussions about surveillance, diagnosis, environmental changes, and health system preparedness(1).

Oropouche is a disease caused by an arbovirus of the genus *Orthobunyavirus*, of the *Peribunyaviridae* family. *Orthobunyavirus oropoucheense* (OROV) was first identified in Brazil in 1960, from a blood sample from a sloth (*Bradypus tridactylus*) captured during the construction of the Belém-Brasília highway. Since then, isolated cases and outbreaks have been documented in Brazil, primarily in the Amazon region. Cases and outbreaks have also been recorded in other countries in Central and South America.

According to the Ministry of Health, 2024 saw a record number of notifications, prompting the expansion of OROV testing in the public health system. The test was included in the arbovirus panel provided by Central Public Health Laboratories in all states, not just the north. This measure aimed to increase diagnostic sensitivity and enable the clinical differentiation of arboviruses with similar presentations, such as dengue, Chikungunya, and Zika—a routine challenge faced by healthcare professionals, especially in primary care settings(2).

That same year, the Department of Health and Environmental Surveillance also developed the Brazilian National Center for Epidemiological Intelligence and Genomic Surveillance (in Portuguese, *Centro Nacional de Inteligência Epidemiológica e Vigilância Genômica* – CNIE) for disease monitoring and control. The CNIE operates through physical and digital structures equipped with cutting-edge communication, audiovisual, and computing technologies, with a qualified technical and scientific team for integrated analysis of strategic health data, with the creation of Arbovirus Observatory, also including data from Oropouche(3).

Oropouche, while typically self-limiting, can present with significant clinical manifestations, including high fever, severe headache, myalgia, nausea, and, in some cases, neurological signs such as aseptic meningitis. Its spread to densely populated urban areas increases the risk of outbreaks, further straining surveillance, and rapid response systems(4).

Concerns about the reemergence of OROV have spread beyond national borders. In late 2024, the U.S. Centers for Disease Control and Prevention issued a travel health alert(5), highlighting the increase in cases of Oropouche in Brazil, specifically in Espírito Santo, which at that time concentrated around 80% of reported cases of the disease, with a concentration of cases in the municipality of Alfredo Chaves, located in the countryside of the state(3). This international alert places Oropouche on the global radar of emerging arboviruses, alongside established threats such as West Nile virus, Mayaro virus, and Chikungunya.

Several factors have contributed to the increased circulation of OROV, such as intensified climate change, loss of vegetation cover, increased population mobility, and rapid urbanization in ecologically sensitive areas. Changes in temperature and precipitation patterns, in particular, have created more favorable conditions for vector reproduction and expanded geographic distribution(1,6).

Given this scenario, nursing—as a strategic category on the front lines of care, health education, and surveillance—needs to be prepared to recognize compatible clinical signs, guide the population, and appropriately report suspected cases. Furthermore, it must work in coordination with health policies to promote preventive measures, especially in contexts of social and environmental vulnerability. If, in 2024, Brazil recorded 13,801 cases of the disease, according to Arbovirus Observatory(3), in 2025, including data from May, these cases will already total 11,706.

The progress of Oropouche challenges us to strengthen syndromic surveillance, the training of health teams, and the integration between levels of care. It also requires a critical look at the impact of environmental changes on the epidemiological patterns of arboviruses. The COVID-19 pandemic has shown that prevention and response to emerging diseases must be proactive, integrated, and evidence-based.

Oropouche represents more than a new epidemiological challenge: the disease reflects contemporary transformations. Coping with it requires science, surveillance, and, above all, an intersectoral commitment to health and the environment, understanding the inseparability of environmental impacts and their consequences for human health.
