# Supplementary material for: Climate change and challenges for health surveillance in the Oropouche emergency
Source: Rev Bras Enferm. 2025 Oct 27;78(Suppl 3):e78suppl302. doi: 10.1590/0034-7167.202578suppl302 (PMC12563835; doi:10.1590/0034-7167.202578suppl302)
Supplement: Translation

# Translation: Mudança climática e desafios para a vigilância em saúde na emergência do Oropouche

**DOI:** 10.1590/0034-7167.202578suppl302pt

**Authors:** Cintia Lepaus Thomas, Karllian Kerlen Simonelli Soares, João Paulo Cola, Ethel Leonor Noia Maciel

**Affiliations:** I Universidade Federal do Espírito Santo, Laboratório de Epidemiologia. Vitória, Espírito Santo, Brasil; II Prefeitura Municipal de Alfredo Chaves, Secretaria Municipal de Saúde. Alfredo Chaves, Espírito Santo, Brasil; III Universidade Federal do Espírito Santo, Programa de PósGraduação em Saúde Coletiva. Vitória, Espírito Santo, Brasil; IV Secretaria de Estado da Saúde do Espírito Santo. Vitória, Espírito Santo, Brasil; V Universidade Federal do Espírito Santo, Departamento de Enfermagem. Vitória, Espírito Santo, Brasil

O ano de 2024 foi marcado por um expressivo aumento nos casos de Oropouche no Brasil, com notificações disseminadas em estados fora da tradicional área endêmica da Amazônia Legal. O Espírito Santo, assim como outras unidades federativas da região Sudeste, passou a registrar casos autóctones, despertando a atenção de autoridades sanitárias nacionais e internacionais. Essa expansão geográfica do vírus Oropouche, transmitido principalmente pelo inseto *Culicoides paraensis*, levanta importantes discussões sobre vigilância, diagnóstico, mudanças ambientais e preparação do sistema de saúde(1).

O Oropouche é uma doença causada por um arbovírus do gênero *Orthobunyavirus*, da família *Peribunyaviridae*. O *Orthobunyavirus oropoucheense* (OROV) foi identificado pela primeira vez no Brasil em 1960, a partir de uma amostra de sangue de um bicho-preguiça (*Bradypus tridactylus*) capturado durante a construção da rodovia Belém-Brasília. Desde então, casos isolados e surtos foram documentados no Brasil, principalmente nos estados da região Amazônica. Casos e surtos também foram registrados em outros países das Américas Central e do Sul.

De acordo com o Ministério da Saúde, 2024 registrou um número recorde de notificações, o que motivou a ampliação da testagem para OROV na rede pública, com a inclusão do exame no painel de arboviroses disponibilizado pelos Laboratórios Centrais de Saúde Pública em todos os estados, não apenas na região Norte. Essa medida visou aumentar a sensibilidade diagnóstica e permitir a diferenciação clínica de arboviroses com apresentação semelhante, como dengue, Chikungunya e Zika — um desafio rotineiro enfrentado por profissionais de saúde, especialmente na Atenção Primária(2).

Neste mesmo ano, foi também desenvolvido pela Secretaria de Vigilância em Saúde e Ambiente o Centro Nacional de Inteligência Epidemiológica e Vigilância Genômica (CNIE) para monitoramento e controle de doenças. O CNIE opera por meio de estruturas físicas e digitais dotadas de tecnologias de ponta em comunicação, audiovisual e computação, contando com equipe técnica e científica qualificada para análise integrada de dados estratégicos em saúde, com a criação do Observatório de Arboviroses, incluindo também os dados de Oropouche(3).

O Oropouche, embora tipicamente autolimitado, pode cursar com manifestações clínicas importantes, incluindo febre alta, cefaleia intensa, mialgia, náuseas e, em alguns casos, sinais neurológicos, como meningite asséptica. Sua expansão para áreas urbanas densamente povoadas amplia o risco de surtos, tensionando ainda mais os sistemas de vigilância e resposta rápida(4).

A preocupação em torno da reemergência do OROV extrapolou as fronteiras nacionais. No final de 2024, os Centros de Controle e Prevenção de Doenças dos Estados Unidos emitiram um alerta de saúde em viagens (*travel health alert*)(5), destacando o aumento dos casos do Oropouche no Brasil, especificamente no Espírito Santo, que naquele momento concentrava em torno de 80% dos casos notificados da doença, com concentração de casos no município de Alfredo Chaves, localizado no interior do estado(3). Esse alerta internacional posiciona o Oropouche no radar global de arboviroses emergentes, ao lado de ameaças já consolidadas como o vírus do Nilo Ocidental, Mayaro e Chikungunya.

Diversos fatores têm contribuído para o aumento da circulação do OROV, como a intensificação das mudanças climáticas, a perda de cobertura vegetal, a maior mobilidade populacional e a rápida urbanização em áreas ecologicamente sensíveis. As alterações nos padrões de temperatura e precipitação, em especial, vêm criando condições mais favoráveis para reprodução dos vetores e ampliação de sua distribuição geográfica(1,6).

Diante desse cenário, a enfermagem — enquanto categoria estratégica na linha de frente do cuidado, da educação em saúde e da vigilância — precisa estar preparada para reconhecer sinais clínicos compatíveis, orientar a população e notificar adequadamente os casos suspeitos. Além disso, deve atuar de forma articulada com as políticas de saúde para promoção de ações preventivas, especialmente em contextos de vulnerabilidade social e ambiental. Se, no ano de 2024, o Brasil registrou 13.801 casos da doença, segundo o Observatório de Arboviroses(3), em 2025, incluindo os dados do mês de maio, esses casos já somam 11.706.

O avanço do Oropouche nos desafia a reforçar a vigilância sindrômica, a capacitação das equipes de saúde e a integração entre os níveis de atenção. Requer também um olhar crítico sobre o impacto das mudanças ambientais nos padrões epidemiológicos das arboviroses. A pandemia de COVID-19 mostrou que a prevenção e resposta a doenças emergentes devem ser proativas, integradas e baseadas em evidências.

O Oropouche representa mais do que um novo desafio epidemiológico: a doença reflete as transformações contemporâneas. Seu enfrentamento requer ciência, vigilância e, sobretudo, um comprometimento intersetorial com a saúde e o meio ambiente, compreendendo a indissociabilidade entre os impactos ambientais e suas consequências na saúde humana.

# Translation: El cambio climático y los desafíos para la vigilancia sanitaria en la emergencia de Oropouche

**DOI:** 10.1590/0034-7167.202578suppl302es

**Authors:** Cintia Lepaus Thomas, Karllian Kerlen Simonelli Soares, João Paulo Cola, Ethel Leonor Noia Maciel

**Affiliations:** I Universidade Federal do Espírito Santo, Laboratorio de Epidemiología. Vitória, Espírito Santo, Brasil; II Prefeitura Municipal de Alfredo Chaves, Departamento Municipal de Salud. Alfredo Chaves, Espírito Santo, Brasil; III Universidade Federal do Espírito Santo, Programa de Postgrado en Salud Pública. Vitória, Espírito Santo, Brasil; IV Secretaria de Estado da Saúde do Espírito Santo. Vitória, Espírito Santo, Brasil; V Universidade Federal do Espírito Santo, Departamento de Enfermería. Vitória, Espírito Santo, Brasil

El año 2024 se caracterizó por un aumento significativo de casos de Oropouche en Brasil, con reportes generalizados en estados fuera del área endémica tradicional de la Amazonia Legal. Espírito Santo, al igual que otros estados de la región sureste, comenzó a reportar casos autóctonos, lo que atrajo la atención de las autoridades sanitarias nacionales e internacionales. Esta expansión geográfica del virus Oropouche, transmitido principalmente por el insecto *Culicoides paraensis*, plantea importantes debates sobre la vigilancia, el diagnóstico, los cambios ambientales y la preparación del sistema de salud(1).

El Oropouche es una enfermedad causada por un arbovirus del género *Orthobunyavirus*, de la familia *Peribunyaviridae*. El *Orthobunyavirus oropoucheense* (OROV) se identificó por primera vez en Brasil en 1960, a partir de una muestra de sangre de un perezoso (*Bradypus tridactylus*) capturado durante la construcción de la carretera Belém-Brasília. Desde entonces, se han documentado casos aislados y brotes en Brasil, principalmente en la región amazónica. También se han registrado casos y brotes en otros países de América Central y del Sur.

Según el Ministerio de Salud, en 2024 se registró un número récord de notificaciones, lo que impulsó la expansión de las pruebas OROV en el sistema de salud pública. La prueba se incluyó en el panel de arbovirus proporcionado por los Laboratorios Centrales de Salud Pública en todos los estados, no solo en el norte. Esta medida tuvo como objetivo aumentar la sensibilidad diagnóstica y facilitar la diferenciación clínica de arbovirus con presentaciones similares, como el dengue, el Chikunguña y el Zika, un desafío rutinario que enfrentan los profesionales de la salud, especialmente en atención primaria(2).

Ese mismo año, la Secretaría de Salud y Vigilancia Ambiental también desarrolló el Centro Nacional de Inteligencia Epidemiológica y Vigilancia Genómica (en portugués, *Centro Nacional de Inteligência Epidemiológica e Vigilância Genômica –* CNIE) para el monitoreo y control de enfermedades. El CNIE opera mediante estructuras físicas y digitales equipadas con tecnologías de comunicación, audiovisuales e informáticas de vanguardia, con un equipo técnico y científico calificado para el análisis integrado de datos estratégicos de salud, con la creación del Observatorio de Arbovirus, que también incluye datos de Oropouche(3).

El Oropouche, aunque suele ser autolimitado, puede presentar manifestaciones clínicas significativas, como fiebre alta, cefalea intensa, mialgia, náuseas y, en algunos casos, signos neurológicos como meningitis aséptica. Su propagación a zonas urbanas densamente pobladas aumenta el riesgo de brotes, lo que sobrecarga aún más los sistemas de vigilancia y respuesta rápida(4).

La preocupación por el resurgimiento del virus del OROV ha trascendido las fronteras nacionales. A finales de 2024, los Centros para el Control y la Prevención de Enfermedades de EE. UU. emitieron una alerta sanitaria para viajeros (*travel health alert*)(5), destacando el aumento de casos de Oropouche en Brasil, específicamente en Espírito Santo, que en ese momento concentraba alrededor del 80% de los casos reportados de la enfermedad, con una concentración de casos en el municipio de Alfredo Chaves, ubicado en el interior del estado(3). Esta alerta internacional coloca a Oropouche en el radar mundial de arbovirus emergentes, junto con amenazas ya establecidas como el virus del Nilo Occidental, el virus Mayaro y el virus Chikunguña.

Varios factores han contribuido al aumento de la circulación de OROV, como la intensificación del cambio climático, la pérdida de cobertura vegetal, el aumento de la movilidad poblacional y la rápida urbanización en zonas ecológicamente sensibles. En particular, los cambios en los patrones de temperatura y precipitación han creado condiciones más favorables para la reproducción del vector y una mayor distribución geográfica(1,6).

Ante este escenario, la enfermería, como categoría estratégica en la primera línea de atención, educación sanitaria y vigilancia, debe estar preparada para reconocer signos clínicos compatibles, orientar a la población y notificar adecuadamente los casos sospechosos. Además, debe trabajar en coordinación con las políticas sanitarias para promover medidas preventivas, especialmente en contextos de vulnerabilidad social y ambiental. Si en 2024 Brasil registró 13.801 casos de la enfermedad, según el Observatorio de Arbovirus(3), en 2025, incluyendo los datos de mayo, estos casos ya ascenderán a 11.706.

El progreso de Oropouche nos reta a fortalecer la vigilancia sindrómica, la capacitación de los equipos de salud y la integración entre los niveles de atención. También requiere una mirada crítica al impacto de los cambios ambientales en los patrones epidemiológicos de las arbovirosis. La pandemia de COVID-19 ha demostrado que la prevención y la respuesta a las enfermedades emergentes deben ser proactivas, integradas y basadas en la evidencia.

El Oropouche representa más que un nuevo desafío epidemiológico: la enfermedad refleja las transformaciones contemporáneas. Afrontarla requiere ciencia, vigilancia y, sobre todo, un compromiso intersectorial con la salud y el medio ambiente, comprendiendo la inseparabilidad de los impactos ambientales y sus consecuencias para la salud humana.
